# Disseminating evidence-based research on mental health and coping to adolescents facing adversity in Lebanon: a pilot of a psychoeducational comic book ‘Somoud’

**DOI:** 10.1186/s13031-020-00324-7

**Published:** 2020-11-18

**Authors:** Tania Bosqui, Anas Mayya, Liliane Younes, Myriam Claire Baker, Ismat Maktabi Annan

**Affiliations:** 1 Department of Psychology, American University of Beirut, Beirut, 1007202 Lebanon; 2 Beit Atfal Assamoud Family Guidance Centre, Beirut, Lebanon

**Keywords:** Mental health, Wellbeing, Adolescents, Dissemination, Psychoeducation

## Abstract

**Background:**

Despite advances in the evidence base and scaling up of mental health and psychosocial interventions for children and adolescents affected by conflict and adversity, a theory-practice gap remains, with limited translation of research findings to affected communities.

**Methods:**

In order to disseminate findings from a review on mental health and coping for children and adolescents, a psychoeducational comic book ‘Somoud’ was developed and piloted with adolescents and their caregivers in Lebanon, using a qualitative Grounded Theory approach.

**Results:**

In total, 10 participants (5 adolescents, 5 parents), took part in the study. Using Thematic Content Analysis, 6 themes were identified; 1) psychoeducation versus misinterpretation, 2) balancing depth with accessibility, 3) aesthetic appeal, 4) contextual relevance and realism, 5) daily stressors, and 6) parental and social relationships.

**Conclusions:**

The findings demonstrate the importance of cultural and contextual piloting of psychoeducational content, and the potential usefulness and accessibility of a comic book format to disseminate information to adolescents. Data from the pilot was used to inform the development of a new version of ‘Somoud’ for sharing with adolescents in Lebanon. Further research is required to establish the effectiveness of the psychoeducational book as a ‘self-help’ tool, and to further improve knowledge exchange between researchers and communities.

## Background

Research has consistently found worse mental health and a higher prevalence rate of mental disorders in populations affected by war and armed conflict [1], as well in populations facing poverty, inequality, and social injustices [2]. Despite major advances in the evidence-base and scaling up of mental health and psychosocial support (MHPSS) for these populations [3, 4] there remains a large theory-practice gap. This gap is comprised of both the lack of dissemination and application of findings from research into practice [5], and of practice and lived experiences informing research needs and priorities [6].

Systematic reviews of MHPSS and health systems in low and middle income countries or humanitarian contexts have consistently highlighted the poor translation of research findings into practice in the field, and inversely, academics’ poor engagement with local services, communities, and affected populations [5, 7]. This is exacerbated by the measurement of research impact using indirect and delayed outcomes such as publications in academic journals and the distal possibility of improved interventions in the future [8]. The poor direct social impact of research raises ethical concerns about the benefit of research to vulnerable populations. Participatory approaches to working with affected communities suggest that research findings should be directly disseminated to communities, so that knowledge can be immediately used to improve health outcomes [9]. Research suggests that such passages of knowledge exchange can be utilized by communities to challenge existing social power structures [10] and research agendas [6]. Direct and immediate access to health information is particularly relevant in countries and populations where there are major structural barriers in accessing health services [11].

There is evidence that exposure to evidence-based psychological literature, can be effective in reducing symptoms of poor mental health, such as depression and anxiety, and that it is more accessible that clinic-based services. Systematic reviews of ‘bibliotherapy’ or ‘self-help books’ based on evidence-based psychological knowledge can significantly improve mental health [12] and improve outcomes for a range of mental disorders [13]. In children and adolescent populations, bibliotherapy or self-help materials have been less well researched, but existing evidence indicates small to moderate effectiveness for externalizing and internalizing behaviors, and pro-social behavior [14]. However, unlike for adults, no known study has included books based on an evidence-based model, and have rather included books with creative or imaginative content. In addition, books are generally aimed only at children, and not to the parent-child dyad or triad. This is particularly problematic in the context of communities facing adversity, where parental support and relationships have been found to be a strong protective mechanism for child and adolescent mental health [15]. Previous studies have used comic book formats to provide accessible education for low literacy socially disadvantaged groups [16], as part of narrative storytelling in trauma interventions for refugees [17], or to inform the wider public about the impact of sexual abuse on children [18]. However, no known studies have explored the use of comic books to disseminate evidence-based mental health and psychosocial information directly to adolescent conflict-affected populations.

Lebanon is a middle-income country affected by the legacy of a 20 year civil war, a recent popular uprising against political corruption and sectarianism, an unprecedented and catastrophic economic crisis, and the Beirut port disaster that killed or injured thousands of people in August 2020. Lebanon is also home to a government estimated 1.5 million refugees in a total population of around 5.9 million [19]. The majority of refugees come from neighboring Syria, as well as an estimated 450,000 Palestinian refugees [20]. In mid-2020, Lebanon’s unemployment rate soared, with almost half of the population living below the poverty line. Forecasts estimate that this will escalate further if measures to improve the economic and social hardships are not found [21]. Refugees in Lebanon face structural discrimination, poverty, and poor access to health care [22]. Almost 80% of Syrian refugees in Lebanon are currently living below the poverty line, with 58% living in extreme poverty and unable to meet basic survival needs [23]. Children and adolescents in Lebanon have been found to experience high levels of anxiety [24]. Adolescents in particular have been found to have an elevated prevalence rate of common mental disorders compared to the rest of the population [25]. At the same time, the health system in Lebanon is predominantly private, and the majority of vulnerable families have no access to it [26]. Despite this, Lebanon has strong governmental support for improved mental health care. The Ministry of Public Health launched the National Mental Health Program in 2014 in partnership with WHO, UNICEF, and International Medical Corps (IMC). In 2015, a 5-year Mental Health and Substance Use strategy for Lebanon 2015–2020 was launched with the aim of reforming the Mental Health System in line with WHO global action plan for Mental Health 2013–2020. Much has been achieved on many fronts but gaps remain, particularly for refugee and marginalized communities.

There is therefore a high need for access to mental health care and information for adolescents in Lebanon, who are facing a context of complex multi-layered social, political, and economic adversity, and a limited public mental health system. In an attempt to directly disseminate usable and practical public mental health information to vulnerable adolescents and their families in Lebanon, a psychoeducational ‘self-help’ comic book was developed, based on a recent review of the evidence base for MHPSS mechanisms of change [15]. This study aimed to pilot the book in order to explore a) whether the book improves knowledge of mental health and coping for adolescents and their caregivers, b) whether adolescents and their caregivers find the book accessible and understandable, and c) in what ways the tool can be improved.

## Methods

This study aimed to explore the acceptability of a novel psychoeducational dissemination tool, a comic book entitled ‘Somoud,’ with a sample of adolescents facing adversity, and their caregivers, in Lebanon. A qualitative research design was used, drawing on the COREQ guidelines [27]. The study aimed to use findings to develop and improve the book for dissemination and wider testing. The research questions were 1) to what extent do adolescents and their caregivers feel that the book improves their knowledge of common psychological reactions to adversity and positive coping strategies? 2) to what extent do adolescents and their caregivers feel that the book format and content is acceptable, understandable, and likeable? 3) in what ways do adolescents and their caregivers feel the book could be improved in format or content? and 4) what are the areas that children, adolescents, and their caregivers, feel they need to know more about or that the book should address?

### Participants

Participants were included if they were a) adolescents aged 12–16, b) with at least one of their caregivers, c) were currently accessing mental health or psychosocial support, d) and were facing adversity. Adversity was broadly defined to include exposure to armed conflict, displacement, poverty, structural discrimination, and social inequality. Participants were included regardless of nationality, refugee status, or religion. The age range was selected to reflect the reading level of the book, which was aimed at adolescents due to the particular lack of clinical research on this population [28, 29]. Adolescents were excluded if they did not meet the inclusion criteria, if the researcher or clinician was concerned of risk to themselves or others, if their capacity to consent was unclear, or if the child or caregiver did not give assent/consent. In total 6 families were invited to participate, in line with recommendations for grounded theory approaches to qualitative methodology [30].

### Materials

The book, named ‘Somoud’ (صمود, a name and concept meaning *steadfastness* or *strength in adversity* in Arabic), was based on a systematic review of reviews on the mechanisms of change for MHPSS that improve the wellbeing and mental health of children and adolescents affected by war and conflict [15]. The main findings that were supported by moderate to high quality evidence were interwoven into the narrative of the book. These mechanisms include family and caregiver support and relationship strengthening [4], psychoeducation on common psychological reactions [31], stress management skills [32], problem solving [4], altruism [33], validating normal reactions to adversity [33], trauma processing [31], cognitive restructuring [32], and therapeutic rapport [4]. The story centers on an adolescent girl, Somoud, her younger brother Samir (to demonstrate different age-relevant reactions to stress, and for gender representation), and their family, who face an unspecified civil conflict. Somoud suffers from nightmares, anxiety and low mood, while Samir suffers from behavioral problems and bed-wetting. The story follows their journey to understand and improve their symptoms, with the support of family, friends, a teacher and a counsellor. The narrative also seeks to contextualize their reactions in the midst of frightening events. Their journey includes explanations and events related to each of the evidence-based mechanisms. The book was developed and illustrated by the first author TB.

### Procedures

Families were invited to participate through the Beit Atfal Assamoud Family Guidance Center, which is a humanitarian non-governmental organization that provides a range of mental health, medical, and psychosocial services to marginalized communities, particularly within Palestinian refugee camps in Lebanon. Participants already accessing services, who met the inclusion criteria, were given the option by their clinician to participate, up until the threshold sample size using convenience sampling. With the permission of families, potential participants’ contact details were passed on to the research team. A member of the research team (co-author AM) contacted the family by phone to give them more information and schedule a meeting at the center. After full written consent and assent was taken, the book was read to the family, and a semi-structured interview was conducted with the family together. Interviews were conducted in a private room in the center, by a male graduate student in clinical psychology (co-author AM), supported by a female psychology undergraduate (co-author IA) or graduate student (co-author MCB). AM was trained by PI TB in qualitative interviewing and risk management, and role played the story telling and interview questions with IA and MCB. No family gave consent to record the interview, so detailed notes were taken by a second member of the research team. The main reason for not wanting to be recorded was fear about how it may be used. One participant was concerned about giving information to an American university. However, they trusted AM and continued with the interview without being recorded. AM reassured them that working with an American university did not equate to any particular political views, nor would identifiable information be shared with anyone outside of the research team. During interviews, co-author LY, a clinical psychologist at the center, remained available in case of any distress or concerns of risk.

Before ending, participants completed a brief demographic questionnaire (including age, gender, income, education, and literacy). Refreshments were provided during the interview, and participants were given 15,000 LBP (around $8–10 at the time of data collection) to cover transport costs. Adolescents were given a copy of the book and a set of coloring crayons. AM reported his experience and reflections to PI TB at the end of every interview. This was used to guide the questions and approach for the next interview, informed by a grounded theory approach to qualitative interviewing [34]. It was also used to discuss emerging themes and data saturation. A broadly defined topic guide was applied based on the aims of the study, but the approach and direction of questioning evolved throughout the interviews. Interviews lasted an average of 90 min. All families were seen once in person, with one follow-up phone call. The follow-up provided an opportunity for the team to reflect on emerging themes from the first interview, and then to ask additional questions to clarify points or to fill gaps in understanding.

### Data analysis

The detailed field notes were first translated from Arabic to English and cross-checked by the two members of the research team present in the interview, and any discrepancies discussed and addressed. Thematic Content Analysis [35] was used to analyze the interview notes, which is rooted in grounded theory and has been used extensively in public health research [36]. The broad structure of the analysis was linked to the aims and topic guides (improving knowledge, format, improvements, and additions). Notes were then read and re-read with open coding for emerging themes, in order to build an initial coding framework within this structure. The coded notes were then re-read and re-arranged, with overlapping codes amalgamated into core themes. Finally, a summary of each theme was drafted with diverse and representative quotes chosen to illustrate the theme, identified by role in the family (adolescent, father or mother), and their age. This process was completed by PI TB and cross-checked by co-author AM. A summary of the themes was presented to participants and final edits made, in line with Creswell’s [37] recommendations for qualitative verification.

## Results

In total 8 families were screened as eligible for participation, 2 of these were excluded (one as the children in the family were all under 12 years old, and the other as the adolescent in the family had unclear capacity to give assent due to an intellectual disability), and 2 of these withdrew after scheduling the interview due to the onset of lockdown measures associated with the COVID-19 pandemic. They were also unable to take part by phone or online due to limited access to technology, electricity, and internet. As the research team agreed we were nearing data saturation, and that lockdown measures would last at least 3 months, we ended the data collection prematurely. Therefore, a total of 4 families took part in the study, comprised of 10 participants. Interviews were conducted between January and February 2020. Four of the five children included were male, and participants had a mean age of 14.4 (SD 1.34). Characteristics of the families are presented in Table 1.

**Table 1:** Demographic characteristics

Age of index child (mean SD)	14.4 (1.34)
Gender of index child (%)	
Female	20%
Male	80%
Families where both parents attended (%)	25%
Family income (%)	
25,000 LBP a day ($17)	25%
‘Unstable’	75%
Families living in a camp (%)	75%
Parents highest education (%)	
Baccalaureate	25%
Grade 9 or less	75%
Literacy (% range of adults and children who can read and write within the household)	40–100%

From the detailed notes of interviews with the included 10 participants, 6 themes were identified; 1) psychoeducation versus misinterpretation, 2) balancing depth with accessibility, 3) aesthetic appeal, 4) contextual relevance and realism, 5) daily stressors, and 6) parental and social relationships. In addition, the reflections of the research team after interviews was amalgamated and presented under interviewer reflections.

### Improving knowledge

#### Theme 1: Psychoeducation versus misinterpretation

Adolescents and their caregivers consistently highlighted that the book was helpful in providing ways to manage anxiety, especially in the context of uncertainty and disadvantage. They also felt it connected with the need to share with family and friends, and seek support from others. This was particularly favored by parents, who reported feeling their children do not share much with them.

Adolescent 1, 13 years old: ‘Breathing slowly helps you calm down’

Mother 1; ‘I liked how the book emphasized communication. The parents and the doctor helped the child get over the problem. Kids don’t tell parents about their fear, or about school. It’s really important to sit with your child and understand their fears.’

Mother 1: ‘The nightmare part was good; learning to understand what caused it and what happened before it.’

Adolescent 3, 15 years old: ‘It gives an idea about fear.’

Mother 3: ‘The idea is to face your fear to get rid of it.’

Mother 1: ‘I liked the fact that the book mentioned a series of problems in the girl’s life. It showed that life problems never stop, you have to keep dealing with them.’

However, both adolescents and parents struggled to extrapolate meaning from some of the metaphors and images used in the book, with children in particular taking messages literally. For example, the clinically commonly used analogy of doing laundry as a metaphor for trauma processing, was often understood by children as the need to keep up with chores. The analogy of the impossibility of *not* thinking about ‘an elephant on roller-skates’ (amended from the commonly used ‘don’t think about a pink elephant’) as an analogy for the negative consequences of thought suppression, was understood by some families as the need to *not* think about difficult memories, which is the opposite of the intended meaning.

Mother 3: ‘I like the idea of laundry, there’s a lot of work. I relate to it [laughter in the room]’

Interviewer: ‘What did you understand from the elephant on the wheels?’

Adolescent 3, 15 years old: ‘Think of something funny.’

Mother 3: ‘It’s rhetorical. Don’t make a big deal out of the dream. Even big worries or difficulties go away. They will get resolved… Humor can help in tense situations, I do that with my husband when we fight.’

Interviewer: ‘What did you get out of the laundry part?’

Adolescent 3, 13 years old: ‘The laundry: do it and fold it.’

Mother 3: ‘The laundry stands for something else: I do my work step by step, I become organized.’

Adolescent 3, 15 years old: ‘There’s an idea but it’s unclear.’

Interviewer: ‘What did you understand of the part about the elephant on wheels?’

Adolescent 4, 15 years old: ‘You can avoid thinking about the shark or the nightmare.’

### Format

#### Theme 2: balancing depth with accessibility

The comic book format was liked by families, and kept adolescents engaged in the story. Most families were literate enough to follow the story as it was being read to them, but reported that children would struggle to read and understand the book without help from an adult. There were divided opinions about how to remedy this, with some parents suggesting more explanations and that the book can then be explained by the parent to the child, and others suggesting that the book should keep its format to remain accessible to children.

Father 1: ‘We can help her understand it [the laundry analogy], and later discuss it.’

Mother 1: ‘It’s more beneficial to read it together and discuss it aloud together. Discussing the story after will clarify some ideas.’

Mother 3: ‘Maybe reduce the photos.’

Adolescent 3, 13 years old: ‘No.’

Mother 3: ‘I want more words.’

Father 1: ‘I also think the book should be more simplified.’

#### Theme 3: aesthetic appeal

Families also commented on the appeal of the pictures and language in the book. They suggested splitting and organizing content differently, and reframing the text, for example to avoid repeatedly using the word ‘fear.’ Families indicated that the aesthetic appeal was important for engagement in the story.

Mother 3: ‘There was a section where “fear” was repeated; it made the story weaker. You can put it like this: عندما تو اجهین مخاو فك یقل الخو ف في المر ة القادمة التي یُثار فیهاشعور ك [when you face your fear, it will diminish the next time you feel it]’

Adolescent 3, 15 years old: ‘You can split the second part into two: one with the teacher and another with the counselor.’

### Improvements

#### Theme 4: contextual relevance and realism

Families gave consistently helpful feedback about ways to address issues with the book content. In particular, the setting and experiences of the family in the book were reported to be more relevant to older generations, who faced active war and armed conflict. Most of the adolescents interviewed were from Palestinian camps, and were born in Lebanon so were exposed more to poverty and disadvantage than active conflict or war.

Father 1: ‘Somoud’s pain is temporary, ours was much more intense. We lived through the Israeli raids. There were almost 62 airstrikes per minute. We had no time to move the bodies off the streets. We developed crocodile skin to adapt and become immune.’

Interviewer: ‘Do you think of adaptation as a positive thing?’

Father 1: ‘It is a good thing because it helps you live, but it makes a part of you die.’

Father 1: ‘The main problem with children lies in their environment... They have no place to play at [sic] and release their energy. No place at all. I cannot take my children out to play once a week. The child cannot let out his energy in a positive way. The harder problem is that you cannot change your child’s environment. I understand what my environment is like, but I cannot extract my child out of it.’

#### Theme 5: daily stressors

Families were concerned with chronic stress and uncertainty, social injustice, discrimination, bullying, parental fighting, and financial insecurity, and the need for children to be resilient and responsible to withstand these difficulties.

Father 1: ‘The camp is divided into districts with some safer than others. I know where we stand inside the camp, but, at the end of the day, we’re still inside the camp.’

Interviewer: ‘What other topics should be added to the book?’

Adolescent 4, 15 years old: ‘Getting bullied.’

Interviewer: ‘okay, by who?’

Adolescent 4, 15 years old: ‘Relatives, school children, even siblings!’

Father 1: ‘The lesson that I want to be taught to children: You have to adapt to tough situations. I have seen so many tragedies in the previous wars, but I learned to adapt to them…you should face your fears; confrontation is more important than breathing [exercises]. The book should focus more on teaching the child not to let the fear live with them.’

Father 1: ‘Trauma/shock (صدمة) is another problem children face. I want my child to be able to differentiate between my death in the camp by a random drug dealer and my death in the South of Lebanon in a protest for the Palestinian cause.’

Interviewer: ‘I’m not sure I understand what you mean?’

Father 1: ‘Life goes on regardless of what happens.’

### Additions

#### Theme 6: parental and social relationships

Many families described the need to include the parental struggle, and the importance of both parental and wider community relationship strengthening, in helping adolescents. Some adolescents also added the stressor of parental fights and family discord as challenging for their mental health and wellbeing.

Father 1: ‘The story needs to address the problems of the parents as well; that is not novel, that this is something your parents and their parents have also lived through. The father is doing all he can.’

Mother 1: ‘The kids are stronger than before, but friendships and social bonds are decreasing lately. Children can’t seem to find enough friends. They’re even worried about the cohesion of their own family. People only act for self-interest.’

Mother 3: ‘Parent’s anger is an issue.’

Adolescent 3, 15 years old: ‘How it affects the children.’

Mother 3: ‘How to resolve fights and control one’s emotions, such as by going out or revising one self [sic].’

### Interview reflections

The interview reflections predominantly concerned the cultural and contextual response of the story and content of the book. Overall, the interviewer felt that concepts of talking to others, facing fears, and social support were culturally acceptable and well received. The use of a nightmare as the hook of the storyline was also thought to be helpful, given its cultural and religious significance. Dreams were found to be an accessible way to talk about mental states, and a helpful way to explore how nightmares can signify emotional distress in the same way that pain can signify physical injury. However, some families seemed to understand facing fears within the narrative of bravery vs cowardice, which inferred shame on those who feel fear or who avoid anxiety-provoking situations. Whilst facing fear is the backbone of psychoeducation on anxiety, shaming is likely to be harmful, and psychoeducation may therefore need to explicitly clarify the difference between anxiety and cowardice, in order to reduce shame and promote adaptive coping. The need to normalize avoidance, as well as nightmares and anxiety, as a core part of the story was felt to be important. Given the range of literacy levels within families, balanced with the need to provide detailed explanations, the interviewer suggested a solution to improve access across families. The solution was to maintain the comic book format, but integrate more explanations for parents, encouragement for children and parents to read together, and to provide audio recordings of the book.

## Discussion

This study aimed to pilot a psychoeducational comic book, based on moderate to high quality evidence on mental health and psychosocial support intervention’s mechanisms of change, with adolescents facing adversity, and their families, in Lebanon. The findings indicate that adolescents and their caregivers found the content helpful to learn about common psychological reactions and ways to cope, and that the format of a comic book was engaging. However, findings also showed that some of the metaphors used in the book to explain psychological processes were often misunderstood, to the point of being perceived as the opposite of the intended meaning. Reflections from the lead interviewer highlighted how different cultural norms and references may have played a role in these perceptions. The interviewer suggested that the narrative needs to anticipate and address culturally-informed unhelpful beliefs or interpretations of the story (such as ‘if you don’t face your fear you are a coward’), and capitalize on helpful ones (such as ‘talking helps’). This is in line with literature that criticizes ‘culturally erroneous attempts’ to import and implement MHPSS content from Western and non-conflict affected populations without adequate consultation, adaptation, and testing in other populations and contexts [38]. It demonstrates how easily conceptual meaning can be lost, and how learning from this pilot is essential to adapt the content of the book ‘Somoud,’ and correct errors, to first and foremost do no harm. Presenting content with clearer explanations and with less or different metaphors, is required for ‘Somoud’ to effectively convey the messages in the book.

The context of the story and narrative was also seen by participating families to be more relevant for past experiences of active war, whereas included adolescents were more troubled by daily stressors related to poverty, family discord, bullying, oppression and structural discrimination. These findings are supported by past research that has found a tendency to neglect addressing daily stressors in conflict affected populations [39], and an assumption of war trauma being the priority problem of all refugee families [40]. To address this, psychoeducational content of the book needs to address this broader context, and the associated daily and systemic stressors. Unfortunately, mechanisms related to wide family and community interventions, and to basic needs like safety and play, are poorly represented in research. The systematic review of reviews on which ‘Somoud’ is based found low quality evidence for mechanisms at this level, predominantly based on case studies and clinical experience [15]. Reviews of MHPSS have consistently highlighted a lack of research on systemic and community interventions [4, 31, 41], and this gap was well highlighted by our participating families. They clearly noticed the lack of inclusion of structural, systemic and contextual challenges that are so damaging to the mental health of both parents and children. Research has found strong links between experiences of oppression, injustice, discrimination and inequality on mental health [2] but these risk factors have been poorly integrated into MHPSS intervention research, limiting psychoeducation to individual processes and coping strategies. For ‘Somoud’ to be an effective tool that addresses the concerns and challenges of adolescents in Lebanon, it must attempt to include and address these contextual and systemic adversities.

Finally, the importance of family and relationships in supporting the mental health and wellbeing of adolescents was consistently reported across families, above and beyond what was already included in the book. This importance is supported by literature on protective factors for adolescents in low and middle income countries and conflict-affected populations around the world, which has found that supportive relationships [42], secure attachments [43], social support [44], community integration [45], consistent parenting [46], and family social support [47] are strong protective factors. These are ways in which the impact of socio-political and economic disasters on the mental health of adolescents may be mitigated, through their support network at the family, peer, and community levels. Their inclusion in psychoeducational material is therefore paramount, and should be made a key message of ‘Somoud.’ Integration of family systems mechanisms into MHPSS interventions and research would go a long way to closing the practice-theory gap, to both improve the evidence-base for family systemic MHPSS intervention effectiveness and mechanisms, and to respond to the clear message coming from clinicians in the field and affected populations themselves.

Based on the findings of this study, a new version of ‘Somoud’ was developed that broadens out the families experiences to include daily stressors and systemic adversities, with clear explanations of concepts and locally relevant metaphors, and a focus on family and community. The book encourages joint reading, with downloadable audio voice-overs, for parents and adolescents to engage with together, whatever their literacy levels. In this version of the book, the frame of the story is the parent, child and sibling relationships, and their connection to their community. They face multi-level adversities, and the psychoeducation is heavy in the normalization of distress reactions in this context. Underlying mechanisms of relationship strengthening, cognitive restructuring, stress management, and problem solving remain but are explored together as a family. The book was also illustrated by a local graphic artist and illustrator, with references from local streets and people to reflect and represent adolescents in Lebanon. This new version will be made available to adolescents accessing the Beit Atfal Assamoud Family Guidance Center in Lebanon, and is available on request from the PI TB.

### Strengths and limitations

This study is the first known study to pilot the use of a psychoeducational comic book to disseminate evidence-based findings on mental health and coping for adolescents affected by conflict. It benefits from in-depth interviews with parents and adolescents, affected by multi-level conflicts and adversities. The study is limited however by the need to take notes instead of record, a smaller sample size than expected due to lockdown measures during the COVID-19 pandemic, and by an unrepresentative population in Lebanon. Participants were all Palestinian, and their views and perceptions may collectively differ from that of other communities in Lebanon. Additionally, the sample had a gender bias (more male adolescents, and more female caregivers) and a potential bias through the recruitment of families who were already willing and able to access mental health care. Further research is needed to test ‘Somoud’ in different communities in Lebanon, and on a larger scale.

## Conclusions

This pilot of the psychoeducational comic book ‘Somoud’ provides preliminary evidence of the usability and acceptability of using comic books to disseminate evidence-based research on mental health and coping for adolescents affected by conflict and adversity. Families highlighted the need for the inclusion of daily stressors and systemic protective factors, with clear and culturally relevant metaphors or explanations. The new version of ‘Somoud’ based on the pilot therefore incorporates all of these changes, but needs to be shared and tested with different populations and on a larger scale before its effectiveness as a self-help guide can be established. The pilot was also an attempt to contribute to addressing the gap between academia and affected populations, by directly disseminating evidence-based research to families and adolescents, and by listening and acting on their feedback for the development of the tool itself.

## Data Availability

The datasets used and/or analyzed during the current study are available from the corresponding author on reasonable request.

## References

[1] Charlson F, van Ommeren M, Flaxman A, Cornett J, Whiteford H, Saxena S (2019). New WHO prevalence estimates of mental disorders in conflict settings: a systematic review and meta-analysis. Lancet..

[2] Lund C, Breen A, Flisher AJ, Kakuma R, Corrigall J, Joska JA, Swartz L, Patel V (2010). Poverty and common mental disorders in low and middle income countries: a systematic review. Soc Sci Med.

[3] Budosan B, Mahoney J, Campos Dorego W, Aziz S, Rafnasavapathipillai K (2020). Three models of scaling up mental healthcare post-disaster: common challenges. Intervention..

[4] Jordans MJ, Pigott H, Tol WA (2016). Interventions for children affected by armed conflict: A systematic review of mental Health and psychosocial support in low- and middle-income countries. Curr Psychiatry Rep.

[5] Tol WA, Barbui C, Galappatti A, Silove D, Betancourt TS, Souza RS, Golaz A, van Ommeren M (2011). Mental health and psychosocial support in humanitarian settings: linking practice and research. Lancet..

[6] Raanaas RK, Bjøntegaard HÖ, Shaw L (2020). A scoping review of participatory action research to promote mental health and resilience in youth and adolescents. Adolescent Research Review.

[7] Greenhalgh T, Fahy N (2015). Research impact in the community-based health sciences: an analysis of 162 case studies from the 2014 UK research excellence framework. BMC Med.

[8] Greenhalgh T, Raferty J, Hanney S, Glover M (2016). Research impact: a narrative review. BMC Med.

[9] Ellis BH, Kia-Keating M, Yusuf AA, Lincoln A, Nur A (2007). Ethical research in refugee communities and the use of community participatory methods. Transcultural Psychiatry..

[10] Atallah DG, Shapiro ER, Al-Azraq N, Qaisi Y, Suyemoto KL (2018). Decolonizing qualitative research through transformative community engagement: critical investigation of resilience with Palestinian refugees in the West Bank. Qual Res Psychol.

[11] WHO (2017). WHO-AIMS report on mental health system in Lebanon.

[12] Gualano MR, Bert F, Martorana M, Voglino G, Andriolo V, Thomas R, Gramaglia C, Zeppegno P, Siliquini R (2017). The long-term effects of bibliotherapy in depression treatment: systematic review of randomized clinical trials. Clin Psychol Rev.

[13] Cuijpers P, Donker T, van Straten A, Li J, Anderson G (2010). Is guided self-help as effective as face-to-face psychotherapy for depression and anxiety disorders? A systematic review and meta-analysis of comparative outcome studies. Psychol Med.

[14] Montgomery P, Maunders K (2015). The effectiveness of creative bibliotherapy for internalizing, externalizing, and prosocial behaviors in children: a systematic review. Child Youth Serv Rev.

[15] Bosqui T, Marshoud B (2018). Mechanisms of change for interventions aimed at improving the wellbeing, mental health and resilience of children and adolescents affected by war and armed conflict: a systematic review of reviews. Confl Heal.

[16] Bitz M (2018). The comic book project: forging alternative pathways to literacy. J Adolesc Adult Lit.

[17] Moore T (2017). Strengths-based narrative storytelling as therapeutic intervention for refugees in Greece. World Federation of Occupational Therapists Bulletin.

[18] Schulz M, Ganzevoort RR, Sremac S (2018). Traumics: the church and trauma in comic book format. Trauma and lived religion.

[19] UNHCR. Lebanon. 2020. http://reporting.unhcr.org/node/2520. Accessed 22 Apr 2020.

[20] UNWRA. Profiling the vulnerability of Palestine refugees from Syria living in Lebanon: United Nations Relief and Works Agency for Palestine Refugees in the Near East; 2015.

[21] World Bank (2020). World Bank: Lebanon is in the midst of economic.

[22] Oxfam (2017). Poverty, inequality and social protection in Lebanon. Oxfam.

[23] UNHCR (2017). Vulnerability assessment of Syrian refugees in Lebanon.

[24] Dimitry L (2012). A systematic review on the mental health of children and adolescents in areas of armed conflict in the Middle East. Child Care Health Dev.

[25] Maalouf F, Ghandour LA, Halabi F, Zeinoun P, Shehan AAS, Tavitan L (2016). Psychiatric disorders among adolescents from Lebanon: prevalence, correlates, and treatment gap. Soc Psychiatry Psychiatr Epidemiol.

[26] Karam E, El Chammay R, Richa S, Naja W, Fayyad J, Ammar W (2016). Lebanon: mental health system reform and the Syrian crisis. BJPsych International.

[27] Tong A, Sainsbury P, Craig J (2007). Consolidated criteria for reporting qualitative research (COREQ): a 32-item checklist for interviews and focus groups. Int J Qual Health Care.

[28] Purgato M, Gross AL, Betancourt T, Bolton P, Bonetto C, Gastaldon C, Barbul C (2018). Focused psychosocial interventions for children in low-resource humanitarian settings: a systematic review and individual participant data meta-analysis. Lancet Glob Health.

[29] UNICEF (2017). Bridging the gap to understand effective interventions for adolescent well-being: an evidence gap map on protection, participation, and financial and material wellbeing in low-and middle-income countries.

[30] Sandelowski M (1995). Sample size in qualitative research. Res Nurs Health.

[31] Betancourt TS, Meyers-Ohki SE, Charrow AP, Tol WA (2013). Interventions for children affected by war: an ecological perspective on psychosocial support and mental health care. Harv Rev Psychiatry.

[32] Peltonen K, Punamäki R (2010). Preventive interventions among children exposed to trauma of armed conflict: a literature review. Aggressive Behav.

[33] Apfel RJ, Simon B (1996). Psychosocial interventions for children of war: the value of a model of resiliency. Medicine Glob Survival.

[34] Tweed A, Charmaz K, Harper AR, Thompson D (2012). Grounded Theory methods for mental health practitioners. Grounded Theory for mental health practitioners.

[35] Newell R, Burnard P (2006). Research for evidence based practice.

[36] Burnard P, Gill P, Stewart K, Treasure E, Chadwick B (2008). Analysing and presenting qualitative data. Br Dent J.

[37] Creswell JW (1998). Qualitative inquiry and research design choosing among five traditions.

[38] Shah SA (2012). Ethical standards for transnational mental health and psychosocial support (MHPSS): do no harm, preventing cross-cultural errors and inviting pushback. Clin Soc Work.

[39] Miller KE, Rasmussen A (2010). War exposure, daily stressors, and mental health in conflict and post-conflict settings: bridging the divide between trauma-focused and psychosocial frameworks. Soc Sci Med.

[40] Summerfield D (2010). Childhood, war, refugeedom and ‘trauma’: three core questions for mental health professionals. Transcultural Psychiatry.

[41] Pedersen GA, Smallegange E, Coetzee A, Hartog K, Turner J, Brown F, MJD J (2019). A systematic review of the evidence for family and parenting interventions in low- and middle-income countries: child and youth mental health outcomes. J Child Fam Stud.

[42] Aba G, Knipprath S, Shahar G (2019). Supportive relationships in children and adolescents facing political violence and mass disasters. Current Psychiatry Reports.

[43] Besser A, Weinberg M, Zeigler-Hill V, Neria Y (2014). Acute symptoms of posttraumatic stress and dissociative experiences among female Israeli civilians exposed to war: the roles of intrapersonal and interpersonal sources of resilience. J Clin Psychol.

[44] Klaric M, Franciskovic T, Klaric B, Grkovic J, Lisica ID, Stevanovic A (2008). Social support and PTSD symptoms in war-traumatized women in Bosnia and Herzegovina. Psychiatr Danub.

[45] O’Donnell DA, Roberts WC (2015). Experiences of violence, perceptions of neighborhood, and psychosocial adjustment among west African refugee youth. International Perspectives in Psychology: Research, Practice, Consultation.

[46] Punamäki R-L, Qouta S, El-Sarraj E (2001). Resiliency factors predicting psychological adjustment after political violence among Palestinian children. Int J Behav Dev.

[47] Shahar G, Henrich CC (2016). Perceived family social support buffers against the effects of exposure to rocket attacks on adolescent depression, aggression, and severe violence. J Fam Psychol.

